# Molecular characterization of environmental Cladosporium species isolated from Iran

**DOI:** 10.29252/cmm.3.1.1

**Published:** 2017-03

**Authors:** I Ghiaie Asl, M Motamedi, GR Shokuhi, N Jalalizand, A Farhang, H Mirhendi

**Affiliations:** 1 Department of Medical Parasitology and Mycology, School of Public Health, Tehran University of Medical Sciences, Tehran, Iran; 2 Department of Medical Parasitology and Mycology, School of Medicine, Shiraz University of Medical Sciences, Shiraz, Iran; 3 Department of Medical Parasitology and Mycology, School of Public Health, National Institute of Public Health, Tehran University of Medical Sciences, Tehran, Iran; 4 Department of Medical Parasitology and Mycology, School of Medicine, Isfahan University of Medical Sciences, Isfahan, Iran

**Keywords:** *Cladosporium* species, Dematiaceous molds, Morphological characteristics, Nuclear ribosomal RNA gene, Sequence analysis

## Abstract

**Background and Purpose:**

*Cladosporium* species are ubiquitous, saprobic, dematiaceous fungi, only infrequently associated with human and animal opportunistic infections*.*

**Materials and Methods:**

Airborne samples were collected using the settle plate method, and soil samples were obtained from a depth of 5-10 cm of the superficial soil layer. Samples were cultured on Sabouraud dextrose agar (SDA) plates, incubated at 25°C, and examined daily for fungal colonies for two to three weeks. Isolates were identified as *Cladosporium *species according to the macroscopic and microscopic criteria. For species differentiation, DNA from 53 isolates was extracted and subjected to amplification of the internal transcribed spacer (ITS) region followed by sequencing.

**Results:**

A total of 270 samples were collected from various environmental sources, of which 79 strains of *Cladosporium* species were isolated. The most frequent species was *C. cladosporioides *(50.6%), followed by *C. iridis *(44.3%), *C. elatum *(2.5%), *C. peranqestum *(1.3%), and *C. alicinum *(1.3%).

**Conclusion:**

The collected data can serve as baseline information for future research and may be useful in the development of preventive and educational strategies.

## Introduction

*Cladosporium* species are among the most common black (dematiaceous) molds [1]. The small conidia of *Cladosporium* species easily spread in large numbers over long distances and represent the most common fungal components isolated from air [2]. The most common *Cladosporium* species are primarily isolated from soil and plant material, where they are frequently encountered as saprobes or secondary invaders on follicular lesions concomitant with other plant pathogenic fungi [3]. *Cladosporium* species are also known to be of common medical relevance inclinical laboratories, being mostly associated with allergic lung mycoses [4], subcutaneous infections [5], and rarely, disseminated infe-ctions [6].

These saprobic species are considered hetero-geneous complexes, composed of several genetically and morphologically distinct species [7]. As of yet, more than 700 species have been identified and described [8]. A wide range of *Cladosporium* species are cosmopolitan, agents of decay, and/or a cause of allergy or even plant or human diseases. The first reports of *Cladosporium* species in Iran dates back to 1939 when Petrak reported *C. herbarium* on *Dianthus orientalis* from Kurdistan, west of Iran [9]. Since then, there have been several sporadic reports of *Cladosporium* species from various substrates in Iran [10, 11]. In all the studies, different species of this genus were identified by the conventional methods based on morphological characteristics. Species belonging to *Cladosporium* are characterized by specific conidiophores, which are erect, straight, or geni-culate and produce abundant branched acropetal chains of olive green to brown conidia with a unique coronate scar structure [12]. However, due to the diversities and similarities among different species, morphological methods are not specific enough, and it appears that a more accurate method is critical to differentiate various members of the genus [13, 14].

In addition to standard procedures, use of state-of-the-art techniques such as polymerase chain reaction (PCR), followed by sequencing of appropriate targets and obtaining data on genetic structure and variation of the fungal populations have important implications for understanding the microbial epidemiology of these fungi [15]. Various DNA-based tech-niques can be applied for genetic study of fungi, of which nucleotide sequence comparison is a reliable approach to systematic molecular study of most fungi. Therefore, we aimed to study the diversity of *Cladosporium* species from environmental sources through DNA sequence analysis*.*

## Materials and Methods

***Sampling***

A total of 270 samples were collected from different sources such as air, soil, grain, fruit, and garbage from different regions, including the Tehran (18.6%) and Isfahan (48.1%) University campus, and public places in the Ardabil Province (33.3%) in Iran. This research was approved by the Ethics Committee of the university (94/290/1596).

Air sampling was performed by the settled plate method, using Sabouraud dextrose agar (SDA, E. Merck, Germany) containing chloramphenicol (100 mg/L) and gentamicin (40 mg/L). Plates were located at different heights on the tree branches and on the ground for 30 min.

For soil sampling, after removing the surface loose litter layer (approximately the top 4 cm), about 15 g of soil was taken from a depth of 5-10 cm of the superficial layer in each location by a spatula, and the collected samples were transferred to the laboratory. An aliquot of 10 g of each soil sample was added to a test tube containing 45 mL of sterile distilled water, mixed for 5 min, and then the suspension was left at room temperature for an hour in order to let the soil precipitate with spores remaining in the supernatant. Subsequently, 15 mL of the supernatant was transferred to another test tube and centrifuged at 2000 rpm for 5 min. Finally, 250 µL of the pellet was added to a Sabouraud dextrose agar plate supplemented with 0.005% chloramphenicol.

All the plates were incubated at 25°C for 2–3 weeks and examined daily based on their dark-colored colony appearance and the conventional morphological methods (i.e., direct microscopic examination and/or slide culture techniques). Colonies suspected to be *Cladosporium* were sub-cultured so as to separate them from other saprophytic molds.

***Molecular investigation***

Total genomic DNA was isolated from each colony using glass bead disruption as previously described [16]. Briefly, fresh colonies (5–10 mm) were transferred to a 1.5-mL tube with 300 mg of glass beads (0.5 mm in diameter), 300 μg of lysis buffer (100 mM Tris- HCl, pH 8; 10 mM EDTA; 100 mM NaCl; 1% sodium dodecyl sulfate (SDS); 2% triton X-100), and 300 μL of phenol-chloroform (1:1). The samples were vortexed vigorously for 5 min and centrifuged for 5 min at 5000 rpm. Then, the supernatant was transferred to a fresh tube in which DNA was extracted with chloroform. An identical volume of isopropanol and a 0.1-volume of 3 M sodium acetate (pH 5.2) were added to the supernatant, and after incubation at -20°C for 30 min, the mixture was centrifuged for 15 min at 12,000 rpm. The precipitate was washed with cold 70% ethanol, dried in air, dissolved in 50 μL of distilled water, and stored at -20°C until use.

ITS1–5.8S–ITS2 rRNA region was amplified using the V9G (5′–TTA CgT CCC TgC CCT TTg TA–3′) and LS266 (5′–gCA TTC CCA AAC AAC TCg ACT C–3′) primers [17]. PCR reactions were performed using 2X PCR Master Mix (Amplicon, Denmark), 25 pmol of each primer, 1 µL of DNA template, and sufficient distilled water to reach a final volume of 25 µL. The following conditions were used for amplification: one cycle at 94°C for 5 min, 30 cycles at 94°C for 30 s, 60°C for 45 s, and 72°C for 45 s, followed by a final extension step at 72°C for 7 min. A negative control (water) was included in all the PCR experiments. Furthermore, 5-μL aliquots of the amplicons were electro-phoresed using a 1.5% agarose gel in Tris-Borate-EDTA buffer (90 mM Tris, 90 mM boric acid, and 2 mM EDTA, pH 8.3) and visualized under ultraviolet irradiation after ethidium bromide staining. A 100-bp DNA ladder was utilized as a molecular size marker.

PCR products from 53 *Cladosporium* strains were purified and sequenced bilaterally by the V9G and LS266 primers using an automated DNA sequencer (ABI PRISM™ ABI-3730 Genetic Analyzer, PE Applied Biosystems, United States). For final identification, the obtained consensus sequences were compared with the *Cladosporium *rRNA barcode database (https://www.ncbi.nlm.nih. gov/pubmed/). Multi-alignment and construction of sequence difference count matrix for edited sequences obtained in the present studyand sequences of clinical strains obtained from GenBank (https://www.ncbi.nlm.nih.gov/pubmed/) were performed using BioEdit software (http://www.mbio. ncsu.edu/bioedit).

## Results

Based on morphological examination, 29.2% (79/270) of the collected samples were identified as *Cladosporium* species. Relative to the number of samples collected in each region, *Cladosporium *was more commonly isolated in Tehran (48%) than in the other two provinces (Esfahan 26.1% and Ardebil 23.3%; Table 1).

Colonies of *Cladosporium *are olive-green toolive-brown with a velvety or powdery appearance. The colonies are diffuse, and the mycelia form mats that rarely grow upwards from the surface of the colony. In cultures, the strains presented microscopic characteristics such as irregular branched conidiophores, brown to olive-brown conidia, coronate scar structures, and conidia in acropetal chains. These morphological features were generally shared among most strains, and we were able to partly identify the *Cladosporium *isolates to species level based on slightly different growth characteristics (Table 1).

**Table 1:** Overview of the *Cladosporium* spp. identified at the three sampling locations included in the present study

**Source (number of collected samples, n)**	**Identified species**	**Number of samples identified by:**	
		**DNA sequencing (%)**	**Morphology (%)**
Campus of Tehran University (n=50)	*Cladosporiumcladosporioides*	14 (17.7%)	9 (17%)
	*Cladosporiumiridis*	10 (12.6%)	5 (9.4%)
Campus of Isfahan University (n=130)	*Cladosporiumcladosporioides*	19 (24%)	19 (35.8%)
	*Cladosporiumiridis*	12 (15.3%)	7 (13.2%)
	*Cladosporiumelatum*	2 (2.5%)	2 (3.8%)
	*Cladosporiumallicinum*	1 (1.3%)	1 (1.9%)
Public places in Ardabil (n=90)	*Cladosporiumiridis*	13 (16.4%)	6 (11.3%)
	*Cladosporiumcladosporioides*	7 (8.9%)	3 (5.7%)
	*Cladosporiumperanqustum*	1 (1.3%)	1 (1.9%)
Total		79 (100%)	53 (100%)

**Figure 1:**
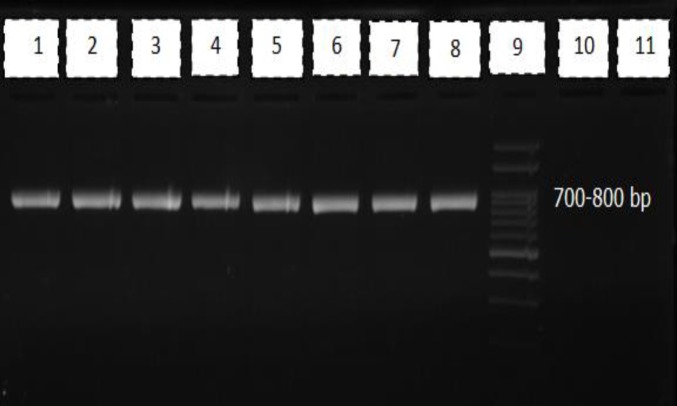
Agarose gel electrophoresis of the ITS1-5.8S-ITS2 rDNA PCR products. Lanes 1-8 are examples of samples, lane 9 is the 100-bp molecular size marker, and lanes 10 and 11 are negative controls

Using a universal fungal rRNA primer pair, a 700–800-bp fragment was successfully amplified from all the isolates, while no PCR amplification was observed in negative controls. Figure 1 demonstrates agarose gel electrophoresis of PCR products amplified from DNA extracted from isolated *Cladosporium* species. The BLAST analysis of the DNA sequences obtained from the 53 * Cladosporium* isolates indicated that *C. cladosporioides *is the most frequently identified species (50.6%), followed by *C. iridis* (44.3%), *C. elatum* (2.5%), as well as *C. allicinum* and *C. peranqustum* (both, 1.3%). The highest species diversity was identified in Esfahan (Table 1).

We found only three DNA sequences reflecting clinical strains of *Cladosporium *spp. in GenBank, all of which belonged to *C. cladosporioides* species (accession numbers: LN8343581, LN8343591 and LN8343601). The lengths of DNA sequences analyzed in this study and those obtained from GenBank were approximately 900 bp and 500 bp, respectively. The sequence alignment of environ-mental (eight random sequences) and clinical (three sequences) strains revealed insignificant divergences, including substitution, insertion/deletion, and gaps throughout the sequences. Sequence inter-species diversity among the 11 strains ranged from 0 to 11 nucleotides.

## Discussion

*Cladosporium* species are found ubiquitously as saprobes in soil and on decayed plant material.

Despite their high prevalence, only a limited number of species have been documented as agentsof human mycotic infections. However, in a comprehensive review on melanized fungi in human diseases, Revankar et al. reported that one of the most common identified agents involved in phaeohyphomycosis is the genus *Cladosporium* [18]. Jafari et al. surveyed the diversity in airborne fungal genera in operation rooms of hospitals in Yazd [19]. Their findings were similar to other reports from Iranian hospitals and indicated that *Cladosporium* is the predominant genus [20, 21]. In spite of its obvious importance, surveys of *Cladosporium* at species level are still scarce.

Members of *Cladosporium* are relatively easy to identify at genus level based on their typical conidiogenous structure. Our results indicated that among the 270 collected samples, 79 (29.3%) were confirmed positive for *Cladosporium*, and the detection rate was comparable to data reported from Kordestan (24.4%) [22] and Malayer (27.2%) [23] provinces of Iran, but higher than that reported in Zanjan (11.9%) [24].

In the present study, five species including*C. cladosporioides*, *C. iridis*, *C. peranqestum*,* C. alicinum*, and *C. elatum* were identified as agents of *Cladosporium* genus in positive culture. During an investigation of *Cladosporium* species asso-ciated with numerous substrates from various localities in Iran during 2011–2013, eight species including *C. delicatulum*, *C. echinulatum*, *C. exile*, *C. macrocarpum*, *C. neriicola*, *C. pannosum*, *C. scabrellum*, and *C. uredinicola* were identified based on morphological characteristics [9]. It is difficult to know to what extent these differences among studies reflect true differences in the epidemiology of this genus. Although variation may reflect real differences observed across the different geographic and climatic regions, the differences may be due to source sampling (air, soil, or other substrates), the method used for identification, and other factors. It is noted that the most prevalent species in each region may change over the course of time.

Morphological identification of *Cladosporium* species is difficult given the high morphological similarity among closely related species. Molecular studies demonstrated that a strategy in which genes are sequenced and the resultant data are analyzed by phylogenetic methods is a robust strategy for fungal species recognition [25]. Sandoval-Denis et al. assessed the diversity of *Cladosporium* species associated with human and animal diseases by analyzing a large set of isolates from clinical specimens by means of DNA sequence data analysis [13]. Since several authors have demonstrated the usefulness of rRNA for species delimitation in *Cladosporium* [26, 27], we used rRNA to identify *Cladosporium* species in this study.

In the present study, no difference in the frequency of identified *Cladosporium* species was observed to depend on the method used (morphological examination vs PCR-sequencing). In both methods, we found that *C. cladosporioides*, the species most frequently cited as being environmentally relevant [8], was strongly represented in our set of isolates. Fresenius first described *C. cladosporioides* in 1850, classifying it in the genus *Penicillium* as *P. cladosporioides*. In 1952, Albertys transferred the species to the genus *Cladosporium* where it remained until today [28]. *C. cladosporioides* has previously been isolated from a pulmonary fungus ball [29], as well as keratitis [30], phaeohyphomycosis [31], and cutaneous and subcutaneous infections [32].

The data exhibited a low degree of polymorphism between environmental and clinical isolates and also a quite low degree of polymorphism within isolates of the same group (clinical or non-clinical group). This might suggest that environmental strains can be a source of human infection. Thus, further studies on DNA sequences derived from clinical isolates is important. This data is comparable to the findings of Haddadi et al., who showed a large amount of variation between two groups by using random amplified polymorphic DNA PCR method [14].

## Conclusion

This study focused on the diversity of *Cladosporium* species isolated from environmental samples by culture and using molecular characterization of the isolates. The most commonly isolated species was *C. cladosporioides*. The collected data can build a foundation for future research and may be useful in the development of preventive and educational strategies. Epide-miological investigations should be performed in multiple areas of the country and compared to data from clinical samples in order to determine the relationship between environmental and clinical strain isolated.
